# Promoting Activity in Geriatric Rehabilitation: A Randomized Controlled Trial of Accelerometry

**DOI:** 10.1371/journal.pone.0160906

**Published:** 2016-08-26

**Authors:** Nancye M. Peel, Sanjoy K. Paul, Ian D. Cameron, Maria Crotty, Susan E. Kurrle, Leonard C. Gray

**Affiliations:** 1 Centre for Research in Geriatric Medicine, The University of Queensland, Brisbane, Australia; 2 Clinical Trials & Biostatistics Unit, QIMR Berghofer Medical Research Institute, Brisbane, Australia; 3 John Walsh Centre for Rehabilitation Research, University of Sydney, Sydney, Australia; 4 Department of Rehabilitation and Aged Care, Flinders University, Adelaide, Australia; 5 Division of Rehabilitation and Aged Care, Hornsby Ku-ring-gai Hospital, Sydney, Australia; Weill Cornell Medical College in Qatar, QATAR

## Abstract

**Background:**

Low activity levels in inpatient rehabilitation are associated with adverse outcomes. The study aimed to test whether activity levels can be increased by the provision of monitored activity data to patients and clinicians in the context of explicit goal setting.

**Methods:**

A randomized controlled trial in three sites in Australia included 255 inpatients aged 60 and older who had a rehabilitation goal to become ambulant. The primary outcome was patients’ walking time measured by accelerometers during the rehabilitation admission. Walking times from accelerometry were made available daily to treating therapists and intervention participants to motivate patients to improve incidental activity levels and reach set goals. For the control group, ‘usual care’ was followed, including the setting of mobility goals; however, for this group, neither staff nor patients received data on walking times to aid the setting of daily walking time targets.

**Results:**

The median daily walking time in the intervention group increased from 10.3 minutes at baseline to 32.1 minutes at day 28, compared with an increase from 9.5 to 26.5 minutes per day in the control group. Subjects in the intervention group had significantly higher non-therapy walking time by about 7 minutes [mean (95% CI): 24.6 (21.7, 27.4)] compared to those in the control group [mean(95% CI): 17.3 (14.4, 20.3)] (p = 0.001).

**Conclusions:**

Daily feedback to patients and therapists using an accelerometer increased walking times during rehabilitation admissions. The results of this study suggest objective monitoring of activity levels could provide clinicians with information on clinically important, mobility-related activities to assist goal setting.

**Trial Registration:**

Australian New Zealand Clinical Trials Registry ACTRN12611000034932 http://www.ANZCTR.org.au/

## Introduction

For frail older people, low levels of mobility during hospitalization are associated with functional decline and deconditioning (decline in muscle strength and bulk as a result of physical inactivity), leading to increased length of stay, post discharge readmission or transfer to permanent residential care [1]. Observational studies [2–4] have shown that activity levels in older people undergoing inpatient rehabilitation are low, with only 7% to 9% of a monitored eight hour period spent walking [3] and 65% of daytime hours either asleep or completely inactive [2]. The factors that contribute to low activity in rehabilitation have not been well studied. One survey of older patients in a post-acute hospital setting found that, whilst attitudes to exercise were generally positive, patients over-estimated the adequacy of their activity levels. They were unsure if they should be doing more exercise and only 11% recalled having been advised to exercise regularly by a health professional [5]. Subjective clinician assessment of the level of patient activity in a rehabilitation setting has also been shown to be inaccurate [6].

Rehabilitation for older people should have specific goals, set in conjunction with the patient, family members and multi-disciplinary team [7]. Goal-setting enhances both the process and outcome of clinical care and is a core practice in rehabilitation [8]. To restore physical function and independence in frail and deconditioned patients, it is important to set measurable, attainable mobility goals and to monitor progress carefully [9]. Monitoring and feedback are essential tenets in health promotion strategies to change behavior, since exercise is significantly influenced by self-efficacy (confidence in one's ability to exercise) and exercise outcome expectations [10]. The setting of activity targets (such as achieving a particular heart rate or step count) has been used to increase levels of physical activity in healthy older people [9]. However, measuring and targeting activity in older patients in rehabilitation settings using models of exercise prescription and monitoring developed for healthy individuals may not be appropriate or accurate [9].

Pedometers, widely used to promote physical activity [11] are inaccurate when assessing step counts as a measure of activity in elderly populations with varying levels of physical dysfunction and gait anomalies [12]. Advances in technology over the past decade have led to the development of wearable devices, such as accelerometers, with potential for continuous ambulatory activity monitoring of older adults in clinical settings [13–16]. Such devices have been used in research studies of gait and balance analysis for falls risk assessment and to detect posture changes for activity monitoring in groups such as amputees, medical and surgical patients, and those with Parkinson’s disease, diabetes, stroke and multiple sclerosis [14].

While the importance of physical activity in the functional recovery of older rehabilitation patients has been recognized [17], questions remain about the optimal methods to promote and monitor activity for frail older people in post-acute hospital settings. Based on the findings of a small feasibility study [18], this study was developed to evaluate the efficacy of using accelerometry to promote activity for older inpatients in rehabilitation settings.

The primary aim was to test whether incidental activity levels can be increased by the provision of objectively measured activity data to patients and clinicians in the context of explicit goal setting. Secondary aims were to explore the effects of increased walking activity (if achieved) on patient outcomes.

## Materials and Methods

### Study Design and Setting

The study design was a parallel group randomized controlled trial, complying with the recommendations from the CONSORT statement [19]. Before selecting this approach, a variety of designs, including matched controlled, before-after and cluster randomized trials were extensively considered, against a set of criteria which included feasibility, degree of contamination, cost and power. The setting was post-acute care Geriatric Rehabilitation Units or Geriatric Evaluation and Management Units with at least a 40 bed capacity, at three Australian sites.

The trial was registered with the Australian New Zealand Clinical Trials Registry (registration number ACTRN12611000034932).

### Participants

Patients admitted to post-acute care rehabilitation who were (1) aged 60 years and older; (2) able to ambulate independently or with supervision/assistance and had a rehabilitation goal to become ambulant within the context of the current admission and (3) expected to have a length of stay of at least two weeks, were eligible to participate. Exclusion criteria were those (1) with lower limb amputation; (2) with delirium or agitated dementia, as documented by the geriatric treating team; or (3) not expected to walk within four weeks of admission.

The study was approved by the Human Research Ethics Committees at each site. Patients gave informed written consent to participate; in the event of incapacity to consent, assent for participation was sought from next of kin or carers.

### Study Protocol

Accelerometers were used to monitor patients’ activity in both the intervention and control groups. For the intervention group, accelerometer data was downloaded daily. Feedback was provided to the intervention participant and their therapists of the previous day’s walking time in numerical and graphical form, showing walking time outside therapy sessions compared with walking target. The treating therapist, in consultation with the patient, set mobility goals, including provisional targets for daily walking time. These goals were reviewed weekly and modified, informed by the accelerometer data, to motivate the patient to improve incidental activity levels outside of therapy sessions and reach set targets. Walking times over a week were summarized in chart form and made available at the weekly case conference. All staff were trained in the use of accelerometry data and asked to encourage patients to meet their activity goals. For the control group, ‘usual care’ was followed, including the setting of mobility goals. However, neither staff nor patients received data on walking times to aid the setting of walking time targets.

The monitoring period was four weeks from date of study entry, unless in the interim, the patient was discharged or unable to continue by virtue of a sudden change in condition which precluded mobility.

### Data Collection and Measures

The primary outcome measure was walking time per day in minutes. Walking time was divided into time within and outside of therapy sessions, based on recorded therapy session times. Secondary outcome measures included lower extremity function and functional status, quality of life, length of monitoring period, and discharge destination measured at study exit and hospital readmissions at 28 day follow-up post study exit.

Walking time was downloaded daily from the accelerometer by the research assistant responsible for activity monitoring. A variation in protocol occurred with change in the activity monitoring device. The accelerometer devices initially used in this study were triaxial ALIVE Heart and Activity Monitors, manufactured by Alive Technologies Pty. Ltd, Ashmore, Queensland, Australia. The device was fitted to a band worn around the waist. Based on validated algorithms [18], the accelerometer measured daily sitting, standing and walking times. A record was kept of periods when the accelerometer was not being worn, mainly at night, and calibrated with times when the signal indicated no activity. Due to difficulties with supply and servicing, the devices were changed part way through the study to ActivPal ^TM^ (PAL Technologies LTD, Glasgow, UK), a validated device [20] which classifies an individual's activity into periods spent sitting or lying, standing and walking. The ActivPal is attached to the front of the mid-thigh with waterproof tape and is capable of continuous monitoring for three to seven days.

A comprehensive geriatric assessment supported by the interRAI Acute Care Post-Acute Care (AC-PAC) instrument was administered *by a* research nurse within three days of entry into the study, at the 14th day and at discharge or exit from the study. The interRAI instrument [21] measures a comprehensive set of items including patient demographics, cognition, mood, functional and mobility status, diagnoses and social support. A number of scales imbedded in interRAI instruments combine single items belonging to a domain, such as personal and instrumental activities of daily living (ADL, IADL), which were used to describe the presence and extent of deficits in that domain [21].

Lower extremity function was assessed by the research nurse at entry into the study, on the 14th day and at discharge or exit from the study, using the Short Physical Performance Battery (SPPB) [22]. The SPPB is a brief, quantitative estimate of future risk for hospitalization and decline in health and function, validated in clinical populations of older adults [23]. The SPPB score is based on timed measures of standing balance, walking speed, and ability to rise from a chair. Each of the three performance measures was assigned a score ranging from 0 to 4, with 4 indicating the highest level of performance and 0 the inability to complete the test. A summary score (range 0–12) is subsequently calculated by adding the three scores [24].

Health-related quality of life was measured using the EQ-5D three level, a concise measure widely used internationally across many different diseases and health states [25]. The output of the instrument is in the form of a utility value (between zero and one) representing quality of life at that point in time. These utilities can be used to estimate quality adjusted life years (QALYs), a common measure of health gain or benefit attributable to an intervention. The EQ-5D was administered by the research nurse at study entry and exit.

A telephone follow-up by the research nurse at 28 days post exit from the study assessed current living arrangements (community, institutional care, died) and adverse outcomes such as readmissions to hospital. According to the approved research protocol, this information could be obtained from the carer, in the event of participant incapacity to respond. All data collection instruments were administered by trained assessors.

### Data Management

An Electronic Case Report Form (eCRF) was developed and validated following Good Clinical Practice standards on Food and Drug Administration approved OpenClinica (www.openclinica.org), a clinical trial software platform for Electronic Data Capture (EDC). The eCRF development and online data capture was managed by a dedicated data manager from the Queensland Clinical Trials and Biostatistics Unit.

### Recruitment and Randomization Procedure

At each site, at admission to post-acute rehabilitation, potentially eligible patients were identified by clinical staff and their names provided to the research assistant who obtained informed consent for participation for those who met eligibility criteria. A random number sequence was generated for the order of group allocation at each site. The randomization codes were generated by the Queensland Clinical Trials and Biostatistics Unit and placed in sealed envelopes. Patients who consented to participate were allocated a unique identification number (ID) and the research assistant opened the sealed envelope for that ID, which contained the randomization code for group allocation. Once opened, the envelope was dated, signed, securely stored and accessed only by the research assistant who was responsible for activity monitoring. A research nurse, trained in the use of assessment instruments and blinded to group allocation, conducted assessments. Rehabilitation staff were not be able to be blinded to the patient’s allocation.

### Statistical Methods

The power analysis for this study was based on accelerometry pilot data of 60 patients [3] with a mean (Standard Deviation (SD)) daily walking time of 45 (51) minutes, correlation coefficient of 0.20 between baseline and 14 day follow-up measurements and a correlation coefficient of 0.60 between the follow-up measurements. Comparative power analyses were conducted for 14 to 20 days of possible repeated measures of daily activities. To observe an increase of activity by at least 15 minutes (33%) in the intervention group, 108 patients were required in each group with 14 days of measurements and 105 patients in each group with 20 days of measurements, with 80% power at two-sided 5% level of significance. During the conduct of the study, activity data were collected for a maximum of 28 days on 255 subjects, thereby significantly increasing the power of the study.

The basic statistics on study parameters were presented by number (%), mean (SD) or median (Interquartile Range (IQR)), as appropriate. Intervention and control groups were compared using Chi-square or Fisher’s Exact tests for categorical variables and t-tests or Mann-Whitney U tests for continuous variables, depending on the distribution of the data, with p value <0.05 taken as the level of significance. The longitudinal trajectories of the daily in-therapy and non-therapy walking time over 28 days were compared between the treatment groups using the generalized estimating equation (GEE) approach, with normal distribution and identity link. The bootstrapped standard errors were obtained, and hence the bootstrapped 95% CI of walking time were presented. The GEE regression model based analyses were weighted by the use of two devices. The changes in the secondary outcome measures at discharge from admission were presented by mean and 95% confidence interval (CI), compared between the treatment groups. All analyses were conducted following the intention-to-treat approach. Analyses were performed using IBM SPSS Version 23.0 (Armonk, NY) and Stata Statistical Software, Release 14 (College Station, TX).

## Results

**Fig 1:**
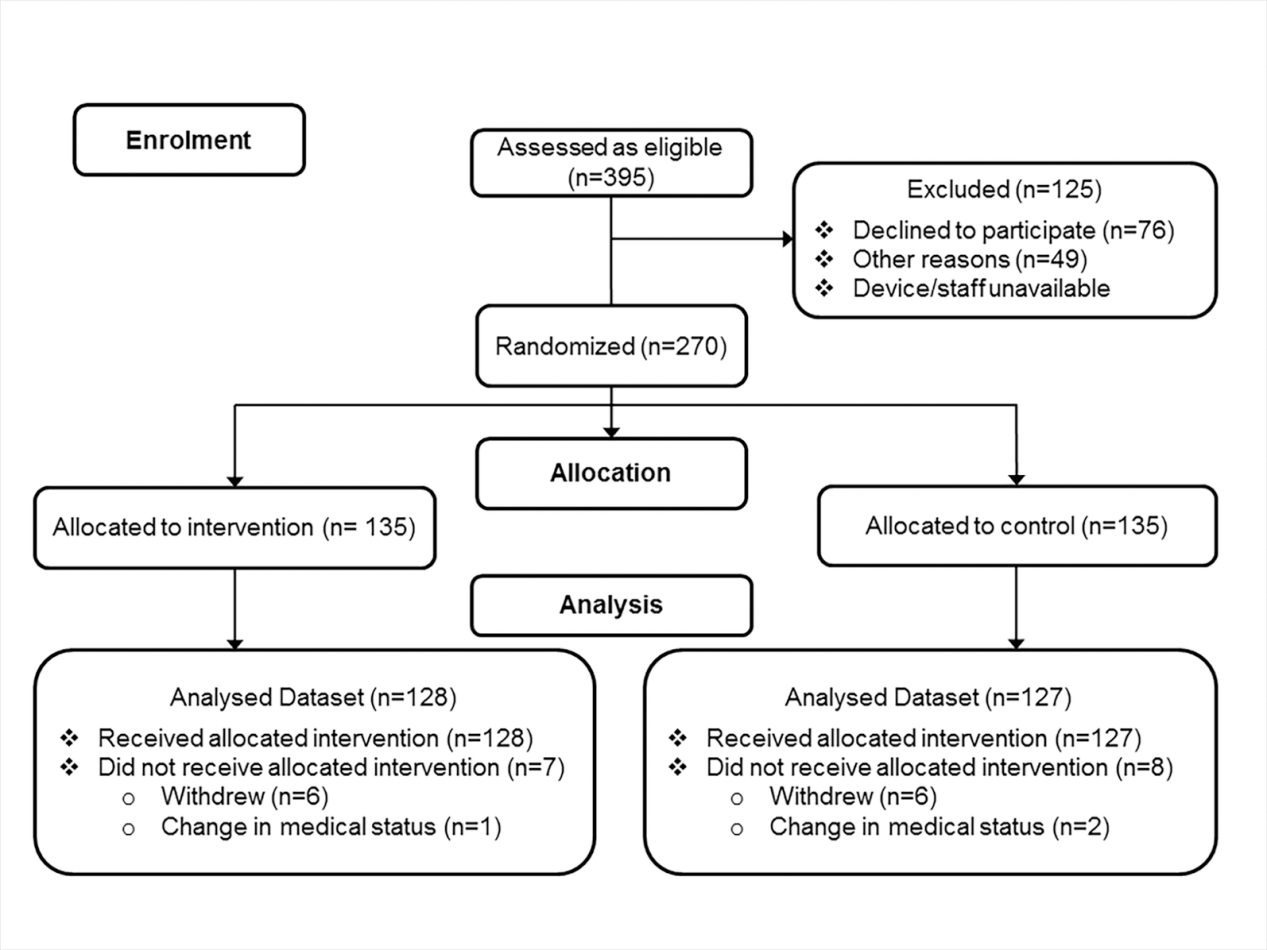
Recruitment Flow Diagram.

A total of 270 subjects (90 from each of the three sites) were randomized equally between the intervention and control group. A total of 128 intervention and 127 control subjects received and continued to follow the study protocol during the course of the study (Fig 1). The median (IQR) length of the monitoring period was 14 (11–18) days, with 6% of patients completing 28 days of monitoring. There was no significant difference in accelerometer device used by treatment group with 141 (55%) of the participants using the ActivPal (71 intervention; 70 control). The daily monitoring period of ActivPal users was 24 hours, as the device was worn continuously. For the ALIVE Heart and Activity Monitor users, the median (IQR) daily hours of monitoring was 8.3 (6.7, 9.6) for the intervention group and 7.3 (6.0, 9.0) for the control group, with no significant difference between treatment groups (p = 0.07).

The mean (SD) age of subjects was 81 (8) years, 42% were male, 16% had BMI above 30 kg/m^2^, and the mean (SD) number of co-morbidities was 8 (4) at randomization. At admission, 84% (n = 215) subjects required supervision or person assistance for walking, and only 16% (n = 40) could walk unsupervised. Gait speed mean (SD) of 0.32 (0.25) m/sec was slow, characteristic of a population in a sub-acute setting with functional dependence and mobility disability [26]. The primary diagnosis in 88 (34.5%) patients was a fracture, most frequently hip fracture (n = 47). Other primary diagnoses were infections (n = 47), including pneumonia and urinary tract infections, neurological conditions (n = 29) including stroke, and cardio-pulmonary conditions (n = 25). There were no significant differences between intervention and control groups on baseline characteristics as shown in Table 1.

**Table 1:** Baseline characteristics of the study subjects by treatment group.

	Intervention	Control
	n = 128	n = 127
Age (years) mean (SD)	81 (9)	82 (8)
Male n(%)	50 (39)	57 (45)
BMI (kg/m^2^) mean (SD)	25.6 (6.7)	24.5 (5.2)
BMI ≥ 30 kg/m^2^ n(%)	25 (20)	16 (13)
Walking- Supervised or Person Assist n(%)	111 (87)	104 (82)
Walking Without Aids n(%)	4 (3)	11 (9)
SPPB median (IQR)^a^	2 (1, 4)	3 (1, 5)
• Gait speed m/s mean (SD)	0.31 (0.24)	0.33 (0.27)
Cognitive Function median (IQR) ^b^	1 (0, 2)	1 (0, 2)
Cognitive Function Score < 2 n(%)^b^	89 (70)	86 (69)
ADL Scale median (IQR)^c^	10 (5, 13)	9 (5, 13)
Number of Co-morbidities mean (SD)	8 (4)	8 (4)
Primary Diagnosis n(%)		
• Fractures	46 (36)	33)
• Infections	24 (19)	18)
• Neurological	14 (11)	12)
• Cardiopulmonary	16 (12)	9 (7)

Notes: Abbreviations: BMI—Body Mass Index; SPPB- Short Physical Performance Battery; ADL—Activities of Daily Living; SD- Standard Deviation; IQR- Interquartile Range

^a^ Based on Short Physical Performance Battery range 0–12 with higher scores indicating better performance

^b^ Based on Cognitive Performance Scale range 0–6 with higher scores indicating greater incapacity

^c^ Based on Activities of Daily Living Scale (Long Form) range 0–28 with higher scores indicating greater dependence

**Fig 2:**
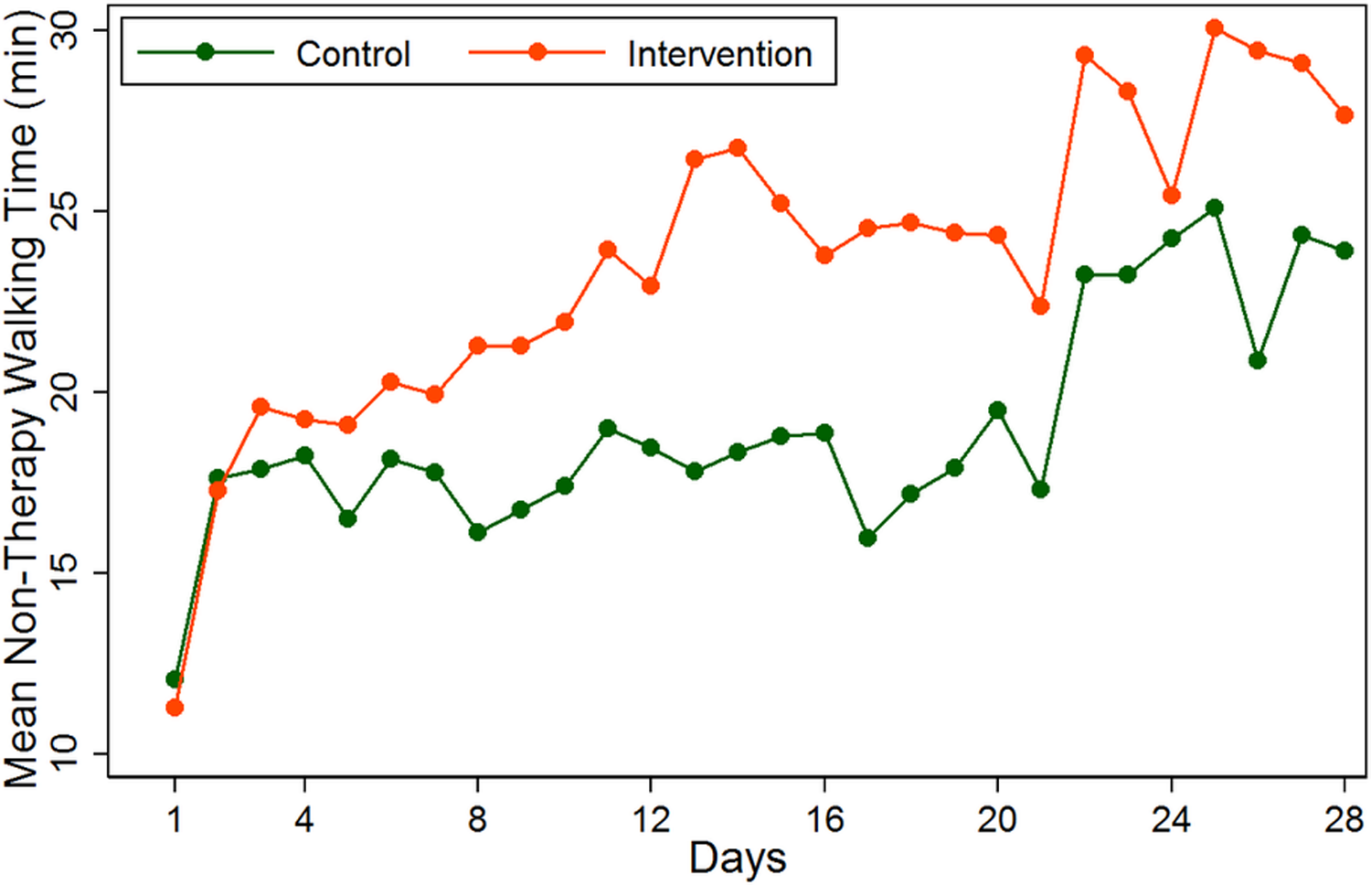
Average daily measures of non-therapy walking time by treatment groups.

**Fig 3:**
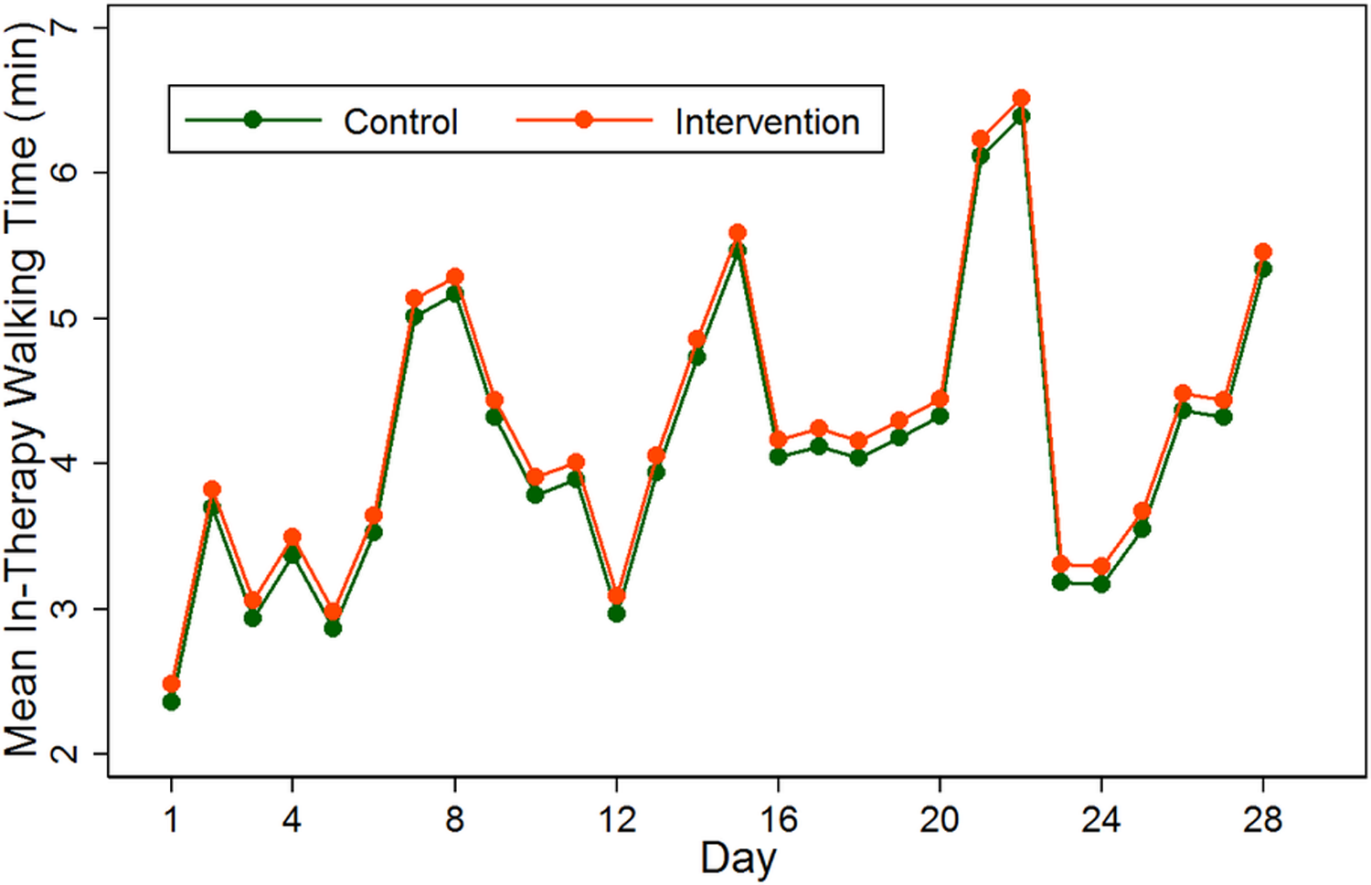
Average daily measures of in-therapy walking time by treatment groups. The measurements are based on GEE regression models, as described in the method section.

The average in-therapy and non-therapy walking time during the 28-day measurement period are presented in Figs 2 and 3. Subjects in the intervention groups had significantly higher non-therapy walking time by about 7 minutes [mean (95% CI): 24.6 (21.7, 27.4)] compared to those in the control group [mean (95% CI): 17.3 (14.4, 20.3)] (p = 0.001, Table 2). The separation of the non-therapy walking time between treatment groups was evident from day 3 post study initiation (Fig 2). There was an observed significant difference in average walking time during therapy (although this is unlikely to be clinically important) (intervention: 4.4 minutes, control: 3.7 minutes, p = 0.021) (Table 2). The median daily walking time in the intervention group increased from 10.3 minutes at baseline to 32.1 minutes at day 28, compared with increase in median walking times from 9.5 to 26.5 minutes per day in the control group.

**Table 2:** Mean (95% CI) of walking time by study group.

	Non-Therapy Walking Time		In-Therapy Walking Time	
	Mean (95% CI)	p	Mean (95% CI)	p
Intervention	24.6 (21.7, 27.4)	0.001	4.4 (4.0, 4.9)	0.021
Control	17.3 (14.4, 20.3)		3.7 (3.2, 4.1)	

Notes: Data are in minutes. Abbreviations: CI- Confidence Interval

In regard to secondary outcomes (Table 3), the median (IQR) length of stay was 23 days (16, 35) and was not different between the treatment groups (p = 0.52). Overall, 16% (n = 41) were discharged to a higher level of care and 7.5% (n = 19) subjects were readmitted within 28 days of discharge. The proportions were not significantly different between the treatment groups (p = 0.63 and p = 0.46 respectively). The improvements in the short physical performance battery (SPPB) and the ADL scale between study entry and exit were not significantly different between the treatment groups. The average levels of individual components of EQ-5D scores were similar between the treatment groups.

**Table 3:** Secondary outcomes at study exit.

Outcome	Intervention	Control	p
Change in SPPB^a^ mean (95% CI)	1.76 (1.33, 2.20)	1.64 (1.21, 2.07)	0.69
• Change in Gait Speed m/s mean (SD)	0.22 (0.21)	0.18 (0.25)	0.12
Change in ADL scale^b^ mean (95% CI)	-5.59 (-6.42, -4.76)	-4.69 (-5.50, -3.88)	0.13
EQ-5D Scores^c^ median (IQR)			
• Mobility	2 (1, 2)	2 (1, 2)	1.00
• Personal Care	1 (1, 2)	1 (1, 2)	1.00
• Usual Activities	2 (1, 2)	2 (1, 2)	1.00
• Pain Discomfort	2 (1, 2)	2 (1, 2)	1.00
• Anxiety / Depression	1 (1, 2)	1 (1, 2)	1.00
Length of monitoring period (days)			
median (IQR)	15 (12, 21)	14 (11, 17)	0.20
Discharged to higher level of care^d^ n (%)	22 (17)	19 (15)	0.63
Readmitted within 28 days n (%)	8 (6)	11 (9)	0.46

Notes

^a^ Based on Short Physical Performance Battery range 0–12 with higher scores indicating better performance

^b^ Based on Activities of Daily Living Scale (Long Form) range 0–28 with higher scores indicating greater dependence

^c^ Based on EQ-5D Items are scored from 1 to 3, corresponding to 3 levels: no problems, some problems, extreme problems respectively.

^d^ Discharged to a higher level of care (eg admitted from community and discharged to residential care)

With the exception of one patient from the control group who withdrew from the study after two hours because they could not tolerate wearing the accelerometer belt, no adverse events were recorded from the use of accelerometers.

## Discussion

Patients in the intervention arm achieved significantly higher non-therapy walking time by 7.6 min/day on average, compared to the control group. Whether this increase in walking time confers a clinically meaningful benefit is debatable, although it is possible that any increase in walking relative to individualised baseline values could confer important health benefits [27]. The prognostic value of physical activity measures such as walking time has not yet been established in a rehabilitation setting, although correlations with physical performance measures such as gait speed have been demonstrated [3, 28]. Remote monitoring using wireless technology is seen as a possible supplementary measure to assess outcomes.

No significant differences were found between treatment groups on secondary outcome measures, although this may be a reflection of the short timeframe of intervention (maximum four weeks) and follow-up (28 days post discharge). Lack of sensitivity to change could also be a factor in not observing differences in the secondary outcome measures, and because a number of physical capacity measures including components of the Short Physical Performance Battery (such as gait speed, chair rise time and balance tests) have floor effects [28].

To our knowledge, this is the first randomized trial that utilized accelerometers as a strategy to improve walking activity for the general population of older patients in a geriatric rehabilitation setting. Two trials to increase walking activity through monitoring and feedback in rehabilitation of stroke patients have recently been published [29, 30]. In contrast to our study, no significant increase in walking time in the intervention group was reported. It was suggested that the rehabilitation environment, patient fatigue and time allocated for other priorities limited opportunities to ambulate more frequently [29, 30].

Comparability of walking times achieved in our study with those in previous studies monitoring activity in geriatric rehabilitation using wearable devices [28–33] are problematic because of differences in population characteristics (including primary diagnosis and acuity), setting (acute, sub-acute or post-acute rehabilitation), period of monitoring and activity measured (eg step count, walking, or ‘uptime’ which includes standing and walking). For example, compared with a baseline median daily walking time of 10 minutes in the current study, daily walking activity varied from 4 minutes in a study of a comparable population referred for rehabilitation therapy [33], to 7 minutes in a study of patients rehabilitated following hip fracture [28] and 23 minutes in moderately impaired elderly stroke patients [31]. Increase in walking time over the duration of rehabilitation shows similar wide variation, depending on diagnostic group and period of monitoring.

While it is widely believed that bed rest and inactivity in hospital are detrimental for mobility and function [34], there are currently no definitive clinical guidelines on optimum physical activity levels for older adults to guide clinicians in the management for older people admitted to rehabilitation [35]. Barriers to mobility during hospitalization of older patients that need to be taken into account in planning successful strategies include health problems, especially weakness, pain, and fatigue; being attached to a medical device such as intravenous drip or catheter; being concerned about falls; and lack of staff to assist with out-of-bed activity [36, 37]. Low mobility among hospitalized older adults has also been attributed to lack of patient motivation [36] and environmental factors such as hospital traffic, noise and clutter that present physical barriers to ambulation [37] and lack of places to go in hospital environs (i.e. patients are not motivated to move) [33].

## Strengths and Limitations

Compared to previous studies, the sample size was large and sufficiently powered to detect changes in the primary outcome measure of difference in walking time between intervention and control groups. Because physical therapy sessions could reflect on time spent walking [33], the walking time in this study was measured for in-therapy and non-therapy times, since the aim of the study was to motivate patients to increase incidental walking activity. Analyses included adjustment for monitoring device.

Accelerometry devices may find difficulty differentiating between walking and standing during very slow walking. This may cause an underestimation of the time spent walking. The absolute percentage error for the ActivPal^TM^ when discriminating between standing and walking is <1% at walking speeds from 0.67–1.56 m/s [38] and 3.5% at 0.45 m/s [39]. Since the participants in this study had a mean gait speed (as measured by the SPPB) of 0.32 m/sec at admission and 0.51 m/sec at discharge, the time spent walking may well be underestimated, and, for this reason, measures of upright time may be more accurate. Changing accelerometry devices during the study could potentially have influenced results, although there was no significant difference in the proportions of participants using each device by treatment group.

Contamination cannot be discounted, since blinding of both therapists and patients was not possible. Both intervention and control groups were aware that activity was being monitored. Such motivation may have minimized possible differences.

## Conclusions

Objective monitoring for the amount of physical activity per day in hospitalized older adults could provide clinicians with information on clinically important, mobility-related activities to assist goal setting. Activity prescription could then be a routine component of the care plan, with professional staff and the patient contributing and responding to the activity plan.

## Supporting Information

S1 File Consort Checklist. (DOCX)

S2 File Accelerometry Trial Study Protocol. (PDF)
