# Supplementary material for: Promoting Activity in Geriatric Rehabilitation: A Randomized Controlled Trial of Accelerometry
Source: PLoS One. 2016 Aug 26;11(8):e0160906. doi: 10.1371/journal.pone.0160906 (PMC5001632; doi:10.1371/journal.pone.0160906)
Supplement: S2 File — (PDF) [file pone.0160906.s002.pdf]

The University of Queensland
AUSTRALIA

CRGM

Centre for Research in Geriatric Medicine
The University of Queensland

Promoting activity for frail aged in post-acute hospital settings: a randomised controlled trial of accelerometry

Clinical Trial Protocol

Contents

General Information 2

Title 2

Funding 2

Project ID 2

Investigators 2

Trial Sites 3

Background Information 4

Rationale 4

References 5

Research Plan 6

Aims 6

Hypothesis 6

Study design and setting 6

Subjects 6

Outcomes 6

Sample size and power analysis 7

Recruitment and randomisation procedure 7

Study procedure 8

Intervention group 8

Control group 8

Data Collection 8

Analysis 9

Database and Statistical Management 9

Ethics 10

Project Management Tasks 10

Companion Documents 11

General Information

Title

Promoting activity for frail aged in post-acute care hospital settings: a randomized controlled trial of accelerometry

Short Title: Promoting activity in geriatric rehabilitation

Funding

National Health and Medical Research Council Project Grant 2011-2013

Project ID

NHMRC ID APP: 1007886
Australian New Zealand Clinical Trials Registry registration number ACTRN12611000034932

Investigators

Project Manager: Dr Nancye Peel
Phone: 07 3176 7402
Fax: 07 3176 6945
Email: n.peel@uq.edu.au
Address: Centre for Research in Geriatric Medicine
The University of Queensland Medical School
Level 2, Building 33
Princess Alexandra Hospital
Ipswich Road
Woolloongabba QLD 4102
www.som.uq.edu.au/research/crgm

Chief Investigators:

Professor Len Gray	Centre for Research in Geriatric Medicine, The University of Qld
Dr Nancye Peel	Centre for Research in Geriatric Medicine, The University of Qld
Professor Ian Cameron	Rehabilitation Studies Unit, The University of Sydney
Dr Mohanraj Karunanithi	The Australian e-Health Research Centre, CSIRO
Assoc. Prof. Sanjoy Paul	Qld Clinical Trials and Biostatistics Centre, The University of QLD

Professor Maria Crotty	Department of Rehabilitation and Aged Care, Flinders University
Assoc. Prof. Susan Kurrle	Rehabilitation and Aged care Services, The University of Sydney

Trial Sites

Geriatric and Rehabilitation Unit, Princess Alexandra Hospital, Woolloongabba, QLD 4102
Rehabilitation Unit, Hornsby Ku-ring-gai Hospital Palmerston Rd Hornsby NSW 2077
Rehabilitation Hospital, 216 Daws Rd, Daw Park SA 5041

Background Information

Rationale

For frail older people, low levels of mobility during hospitalization are associated with functional decline and deconditioning (decline in muscle strength and bulk as a result of physical inactivity), leading to increased length of stay, post discharge readmission or transfer to permanent residential care [1]. While the importance of physical activity in the functional recovery of older rehabilitation patients has been recognised, questions remain about the optimal methods to promote and monitor activity for frail older people in post-acute hospital settings [2].

Observational studies [3,4] have shown that activity levels in older people undergoing in-patient rehabilitation are low. Essential activity (such as toilet visits) accounted for 3.4% of waking hours, whilst self-directed physical activity accounted for only 0.4% of the patient's time. Patients spent 65% of daytime hours either asleep or completely inactive [3]. The factors that contribute to low activity in rehabilitation have not been well studied. One survey of older patients in a post-acute hospital setting found that whilst attitudes to exercise were generally positive, patients over-estimated the adequacy of their activity levels. They were unsure if they should be doing more exercise and frequently stated health problems as a barrier to increased activity. Only 11% recalled having been advised to exercise regularly by a health professional [5].

Rehabilitation for older people should have specific goals, set in conjunction with the patient, family members and multi-disciplinary team [6]. Goal-setting enhances both the process and outcome of clinical care and is a core practice in rehabilitation [7]. To restore physical function and independence in frail and deconditioned patients, it is important to set measurable, attainable mobility goals and to monitor progress carefully [2] . Monitoring and feedback are essential tenets in health promotion strategies to change behaviour, since exercise is significantly influenced by self-efficacy (confidence in one's ability to exercise) and exercise outcome expectations [8]. The setting of activity targets (such as achieving a particular heart rate or step count) has been used to increase levels of physical activity in healthy older people [2]. However, measuring and targeting activity in older patients in rehabilitation settings using models of exercise prescription and monitoring developed for healthy individuals may not be appropriate or accurate [2].

Current methods for prescribing activity for older hospitalised patients are to ask staff to walk patients on a regular basis and monitor what they do. Such walking programs have been successfully implemented in acute care settings, with reported outcomes such as increases in mobility and independence [8]. However such ad hoc programs are expensive, resource intensive and are unlikely to produce a measurable output. Alternatively, we have the opportunity to utilise accelerometry for continuous, unsupervised, objective monitoring of mobility.

Pedometers, widely used to promote physical activity [9] are inaccurate when assessing step counts as a measure of activity in elderly populations with varying levels of physical dysfunction and gait anomalies [10]. Advances in technology over the past decade have led to the development of wearable devices, such

as accelerometers, with potential for continuous ambulatory activity monitoring of older adults in clinical settings [1]. Such devices have been used in research studies of gait and balance analysis for falls risk assessment and to detect posture changes for activity monitoring in groups such as amputees, medical and surgical patients, and those with Parkinson's disease, diabetes, stroke and multiple sclerosis [1].

The study proposed in this protocol is one of a series designed to explore the efficacy of accelerometry in rehabilitation and geriatric assessment services in hospital.

References

1. Brown CJ, Friedkin RJ, Inouye SK. Prevalence and outcomes of low mobility in hospitalized older patients. J Am Geriatr Soc 2004;52(8):1263-70.
2. Dawes H. The role of exercise in rehabilitation. Clin Rehabil 2008;22(10-11):867-70.
3. Patterson F, Blair V, Currie A, Reid W. An investigation into activity levels of older people on a rehabilitation ward: an observational study. Physiotherapy 2005;91(1):28-34.
4. Smith P, Galea M, Woodward M, Said C, Dorevitch M. Physical activity by elderly patients undergoing inpatient rehabilitation is low: an observational study. Aust J Physiother 2008;54(3):209-13.
5. Buttery AK, Martin FC. Knowledge, attitudes and intentions about participation in physical activity of older post-acute hospital inpatients. Physiotherapy 2009;95(3):192-8.
6. Cameron ID, Kurrle SE. 1: Rehabilitation and older people. Med J Aust 2002;177(7):387-91.
7. Wade DT. Goal setting in rehabilitation: an overview of what, why and how. Clin Rehabil 2009;23(4):291-5.
8. Killey B, Watt E. The effect of extra walking on the mobility, independence and exercise self-efficacy of elderly hospital in-patients: a pilot study. Contemp Nurse 2006;22(1):120-33.
9. Bravata DM, Smith-Spangler C, Sundaram V, Gienger AL, Lin N, Lewis R, et al. Using pedometers to increase physical activity and improve health: a systematic review. JAMA 2007;298(19):2296-304.
10. de Bruin ED, Hartmann A, Uebelhart D, Murer K, Zijlstra W. Wearable systems for monitoring mobility-related activities in older people: a systematic review. Clin Rehabil 2008;22(10-11):878-95.

Research Plan

The study proposed here is one of a series designed to explore the efficacy of accelerometry in rehabilitation and geriatric assessment services in hospital.

Aims

The primary aim of this proposal is to test whether activity levels can be increased by the provision of accurate activity data to patients and clinicians in the context of explicit goal setting. Secondary aims are to explore the effects of increased walking activity (if achieved) on patient outcomes and undertake a cost benefit analysis.

Hypothesis

The central hypothesis is that, in post-acute restorative care settings, the provision of activity data obtained by accelerometry, provided to patients and clinicians in the context of explicit goal-setting, will result in a mean increase of 33% in daily walking activity in the intervention group compared with controls offered standard care.

Study design and setting

A parallel group randomised controlled trial will be conducted. The setting will be post-acute care Geriatric Rehabilitation Units or Geriatric Evaluation and Management Units at 3 sites (QLD, NSW, and SA) which have at least a 40 bed capacity.

Subjects

Patients admitted to post acute care rehabilitation who are (1) aged ≥60 years; (2) able to ambulate independently or with supervision, or who have a rehabilitation goal to become ambulant within the context of the current admission; and (3) expected to have a length of stay of at least 2 weeks, will be eligible to participate. Exclusion criteria will be those (1) with lower limb amputation; (2) with delirium/agitated dementia.

Outcomes

The primary outcome measure will be walking time per day collected using the accelerometer. The accelerometer devices used in this study are tri-axial ALIVE Heart and Activity Monitors, manufactured by Alive Technologies Pty. Ltd. The device is 90mm X 40mm X 16mm, weighs 55g with the battery, and measures acceleration in 3 directions using a spring mass system. A single device is fitted in a pouch attached to a belt worn around the waist.

Secondary outcome measures will include lower extremity function and functional status, quality of life (QoL), length of stay, post discharge hospital readmissions or differences in level of care.

Sample size and power analysis

The background information for sample size calculation is based on accelerometry pilot data of 60 patients with a mean (SD) daily walking time of 45 (51) minutes, correlation coefficient of 0.20 between baseline and 14 follow-up measurements and a correlation coefficient of 0.60 between the follow-up measurements. The power analyses are based on 14 and 20 days of possible repeated measures of daily activities. To observe an increase of activity by at least 15 minutes (33%) in the intervention group, 108 patients are required in each group with 14 days of measurements and 105 patients in each group with 20 days of measurements, with 80% power at 5% level of significance.

We will recruit 270 patients over a period of 12 months, allowing for an attrition rate of 20%.
Based on a previous study that mobility activity level is strongly correlated (0.79) with functional independence as measured by the Barthel Index, we hypothesise that an increase in average daily walking time by 15 minutes (33%) would result in clinically significant gains in functional capacity.

Feasibility of recruiting required sample size
Based on a conservative recruitment rate of 2 patients per week at each site, it would take 48 weeks to complete data collection for 90 patients (270 for the 3 sites) based on an average length of stay of 4 weeks. A minimum of 8 accelerometers would be required per site.

Recruitment and randomisation procedure

The site coordinator will explain the study protocol with clinical staff and publicise the study within the rehabilitation wards.

The Research Assessor (RA) will liaise daily with the clinical coordinator and clinical staff to identify potential participants for the study then screen for eligibility. Patients who meet the eligibility criteria will be in either “eligible now” (ready to ambulate with or without supervision) or “potentially eligible in the future” (not ready to mobilise but who have a rehabilitation goal to become ambulant in the context of the current admission”. For those “eligible now” and if the device is available the RA will seek informed consent to participate. For those “potentially eligible in the future” the RA will continue to liaise with clinical staff to determine when the patient is ready to mobilise. As soon as ready to mobilize and the device is available, the RA will seek informed consent to participate using the Ethics approved Patient Information and Consent Forms.

As soon as a patient consents to participate, the RA will allocate a participant ID number- the first two digits will signify the site (01: QLD; 02: NSW; 03: SA) and the following 3 digits are the patient unique ID starting at 001 and consecutively until the recruitment target of 90 patients is reached (ie 090). The RA then gives the participant ID number to the Accelerometry Monitor (AM) who will open the envelope identified by ID

number and allocate to intervention or control group according to the code inside. Randomisation codes will be generated by the Queensland Clinical Trials and Biostatistics Centre. The RA is blinded as to group allocation.

Study procedure

For both groups the AM will show the study participants how to put on the accelerometer belt which they are required to wear from rising to going to bed (and for at least 8 hours) each day over the study period. The participant will remove the accelerometer belt in the evening and when showering. The AM will monitor and record the level of compliance with wearing the accelerometer. In addition the AM will obtain from the treating physiotherapist the daily time spent in therapy sessions.

For each participant, the duration of the study is expected to be 28 days (4 weeks) unless in the interim, the patient is discharged or unable to continue in the study by virtue of a sudden change in condition which precludes mobility. Each weekday morning, the AM will download data from the accelerometers for daily readings of walking time, (and other activity measures).

Intervention group

At commencement, the treating physiotherapist will be asked to discuss with the intervention participants their mobility goals, including provisional targets for daily walking time. These goals will be re-examined and modified, informed by the accelerometer data, to motivate the patient to improve activity levels and reach set targets. **The walking time targets are based on walking time outside of therapy sessions**. Information provided to the treating physiotherapists and the participants will include daily walking time outside therapy as well as daily walking time targets in numerical and graphical form. The targets will be reviewed daily until discharge or 4 weeks, whichever comes first. Walking times over a week will be summarised in chart form and made available at the weekly case conference. Staff will be trained in the use of accelerometry data and be asked to encourage patients to meet their activity goals.

Control group

The control group will have" usual" care that is provided by the rehabilitation staff. However, neither treating staff nor patients in the control will receive data on walking times to aid the setting of walking time targets.

Data Collection

- Walking times will be collected using the accelerometer. The accelerometer will also provide daily sitting, standing and lying times. At night, when the accelerometer is not being worn, it is assumed that the patient is lying down; lying times will be imputed accordingly.
- Lower extremity function will be assessed by the RA at entry into the project, on the 14th day and at discharge or exit from the project, using the Short Physical Performance Battery.
- Change in mobility status (whether walking independently or requiring supervision) will be assessed and recorded weekly by the AM.

- The interRAI AC PAC instrument will be administered by the RA within 3 days of entry into the project, at the 14th day and at discharge or exit from the project.
- The Short Self-Efficacy and Outcome Expectation for Exercise Scales will be administered by the RA at entry and exit from the project.
- Health-related quality of life will be measured using the EQ-5D administered by the RA at study entry and exit, as well as at the 28 day post discharge follow-up.
- A telephone follow-up by the RA at 28 days post exit from the study will assess functional status [Activities of Daily Living (ADLs) and locomotion from the interRAI AC PAC, quality of life (EQ-5D) and adverse outcomes such as readmissions to hospital or changes to a higher level of care (e.g. community to residential aged care].

Analysis

The primary analysis will be based on the repeated daily measures of walking time, compared between the intervention and control groups. Appropriate repeated measure technique will be adopted to analyse this data, depending upon the nature of the distribution of walking time. The changes in the scores related to lower extremity function, EQ-5D and other data between baseline and follow-up will be analysed using appropriate non-parametric techniques. InterRAI generated information will be compared between the groups using appropriate statistical techniques. All analyses will be based on 'Intention to Treat' population.

A formal statistical analysis plan will be in place once the study protocol is finalised and approved. No interim analysis is planned for this study. The data management, statistical analysis and the reporting of the study will strictly follow the standard operation procedures adopted by the Queensland Clinical Trials & Biostatistics Centre.

Economic Analysis

A "with-in trial" economic analysis will be undertaken from the perspective of the healthcare system. The research costs will be excluded from this analysis so that the intervention group (with daily feedback on walking times) can be compared to a usual care group (who receive general discussion around mobility goals). This enables results to be generalised beyond a research setting.

We will collect data to accurately record costs at the patient and ward levels. Cost data will include staff and technician time in applying the accelerometer, downloading and analysing data, and AM time to feedback the previous day's walking achievements to the patient. Ward costs include a national average per diem cost for length of stay and any costs for readmissions during the follow-up period. Equipment costs will include accelerometers, computation and printing. The outcome data (including walking time, length of stay and quality of life) will enable both cost-effectiveness and cost-utility analyses to be performed. Sensitivity analyses will enable these estimates to be adjusted for expected cost reductions associated with increased use (more patients per ward will secure economies of scale) and price reduction as the technology improves.

Database and Statistical Management

The Electronic Case Report Form (eCRF) will be developed following Good Clinical Practice standards on Food and Drug Administration approved OpenClinica® (www.openclinica.org), which is a clinical trial software platform for Electronic Data Capture (EDC). The eCRF development and online data capture will be managed by a dedicated data manager.

The database manager will:

1. Be responsible for database development, validation, eCRF installation and webhosting
2. Ensure the consistency and quality on a continuous basis. She will ensure all measures are imported into the database after a quality check. The accelerometry data will be loaded into the database by the AM.
3. Report recruitment flow monthly.

The project statistician will:

1. Along with the CIs, finalise the statistical analysis plan (SAP) within 6 months after the study starts.
2. Finalise the statistical programming codes for all steps of the analyses, tables and shells by 10 months after the finalisation of SAP.
3. Together with the research team, decide the date for "database lock" following the nature of recruitment and after completion of all data-related checks and queries.
4. Start statistical analysis after receiving analysis ready data sets from the database manager.
5. Provide the final analysis report within 3 months after receiving analysis ready data.
6. Together with the writing committee, start developing manuscripts after the completion of statistical analysis.

Ethics

Prior to commencement of the project, ethics approval was obtained from the Human Research Ethics Committees (HRECs) at the three sites where the project is to be carried out, as well as to the HREC, the University of Queensland. Approval references are as follows: HREC/11/QPAH/043 of 11 March 2011; HREC/11/HAWKE/14 of 1 March 2011; SAFUHREC 012.11 of 24 March 2011; the University of Queensland Medical Research Ethics Committee 2011000476 of 18 April 2011.

Written informed consent will be obtained as a condition of study entry. Cognitive impairment or mental illness will not be grounds for exclusion. Inclusion criteria will be that the patient has the capacity (as assessed by the referring geriatrician, rehabilitation specialist or clinical staff) to give informed consent and understand and be capable of complying with set activity goals. Patient information and consent forms as approved by HRECs will be used for participant recruitment.

Project Management Tasks

**Task**	**Timeframe**
1. Ethics application submitted	By January 2011

2. Research site governance and contracts submitted for approval	By March 2011
3. Recruitment of centres and personnel	By April 2011
4. Develop protocol including data management	By September 2011
5. Site establishment and training of team	By September 2011
6. Trial Intervention	October 2011 to December 2012
7. Data entry	October 2011 to December 2012
8. Data analysis	January 2013 to March 2013
9. Write up and dissemination	April 2013 to June 2013

Companion Documents

THIS PROTOCOL SHOULD BE READ IN CONJUNCTION WITH:

1. Study Guidelines Version 2 01.11.11
2. Open Clinica Manual
